# Nutritional Strategies to Counteract Mitochondrial Dysfunction and NAD+ Deficiency in Human Sarcopenia

**DOI:** 10.1093/geroni/igaa057.2760

**Published:** 2020-12-16

**Authors:** Jerome Feige

**Affiliations:** Nestlé Research, Lausanne, Switzerland

## Abstract

The causes of impaired skeletal muscle mass and strength during aging are well-studied in healthy populations. Less is known on pathological age-related muscle wasting and weakness termed sarcopenia, which directly impacts physical autonomy and survival. We compared genome-wide transcriptional changes of sarcopenia versus age-matched controls in muscle biopsies from 119 older men of different ethnicity. Individuals with sarcopenia demonstrate a prominent transcriptional signature of mitochondrial bioenergetic dysfunction in skeletal muscle, with low PGC-1α/ERRα signalling, and downregulation of oxidative phosphorylation and mitochondrial proteostasis genes. These changes translate functionally into fewer mitochondria, reduced bioenergetic activity, and NAD+ deficiency in sarcopenic muscle. Our results point to mitochondrial homeostasis as a key mediator of pathological muscle aging. Novel nutritional solutions enhancing muscle strength and performance by enhancing mitochondrial function are being tested clinically and will be reviewed. These include activating mitophagy with Urolithin A or restoring NAD+ levels via tryptophane/kynurenine or with nicotinamide riboside.

